# Effect of a Product Containing Xyloglucan and Pea Protein on a Murine Model of Atopic Dermatitis

**DOI:** 10.3390/ijms21103596

**Published:** 2020-05-19

**Authors:** Michela Campolo, Giovanna Casili, Irene Paterniti, Alessia Filippone, Marika Lanza, Alessio Ardizzone, Sarah A Scuderi, Salvatore Cuzzocrea, Emanuela Esposito

**Affiliations:** Department of Chemical, Biological, Pharmaceutical and Environmental Sciences, University of Messina, Viale Ferdinando Stagno D’Alcontres, 31-98166 Messina, Italy; campolom@unime.it (M.C.); gcasili@unime.it (G.C.); ipaterniti@unime.it (I.P.); afilippone@unime.it (A.F.); mlanza@unime.it (M.L.); aleardizzone@unime.it (A.A.); sarascud@outlook.it (S.A.S.); salvator@unime.it (S.C.)

**Keywords:** atopic dermatitis, *staphylococcus aureus*, xyloglucan, pea protein

## Abstract

Atopic dermatitis (AD) is a chronic inflammatory disease of the skin, characterized by dryness and more or less severe itching. The etiology of AD is complex and has not been fully clarified, involving genetic susceptibility, immunological abnormalities, epidermal barrier dysfunction, and environmental factors. Xyloglucan (XG) and pea protein (PP) are two compounds of natural origin characterized by the ability to create a physical barrier that protects mucosae membranes, reducing inflammation. The aim of the present study was to evaluate the potential beneficial effects of XG + PP in both a mouse model of AD and *Staphylococcus aureus* (*S. aureus*) infection- associated AD. Mice were topically treated with 200 μL of 0.5% oxazolone on the dorsal skin three times a week for AD induction. Mice received XG and PP by topical administration 1 h before oxazolone treatment. In *S. aureus* infection-associated AD, to induce a superficial superinfection of the skin, mice were also treated with 5 μL of 10^8^ of a culture of *S. aureus* for 2 weeks; mice superinfected received XG and PP by topical administration 1 h before oxazolone + *S. aureus*. Four weeks later, the skin was removed for histological and biochemical analysis. Our results demonstrated the protective barrier effects of XG and PP characterized by a reduction in histological tissue changes, mastocyte degranulation, and tight junction permeability in the skin following oxazolone treatment. Moreover, XG + PP was able to preserve filaggrin expression, a hallmark of AD. Our data also support the effectiveness of XG + PP to reduce the damage by superinfection post AD induced by *S. aureus*. In conclusion, a future product containing XG and PP could be considered as a potentially interesting approach for the treatment of AD.

## 1. Introduction

Atopic dermatitis (AD) is a skin chronic inflammation characterized by lesions that overlap in relation to the stage of the disease; these induce intense itching and dry skin [1]. Chronic inflammation leads to a decrease in protective epidermal functions, physiological moisturizing factors, and antimicrobial proteins [2].

The etiopathogenesis of AD is still not clear: it could be related to immune abnormalities, genetic susceptibility such as filaggrin mutation, and environmental factors [3]. AD affects 5%–20% of children between ages 3 and 6 months [4]; in most cases, the disease spontaneously improves during puberty, but in up to 10% of cases, it can persist into adulthood [5,6].

Furthermore, the disease usually has a chronic-relapsing course with periods of improvement alternating with more or less severe exacerbations; particularly, bacterial superinfections affected by *S. aureus* and streptococci are common. The treatment of AD is based on the use of moisturizers, topical corticosteroids and immunomodulators, systemic antihistamines and corticosteroids, cyclosprin, and phototherapy [7].

The use of non-pharmacological products to treat AD could be considered a useful and safe alternative to antibiotics and glucocorticoids [8].

Currently, there is a strong interest for a new class of products, which are defined as “mucosal protectors”, such as xyloglucan [9], which forms a bioprotective film in both the intestinal and urinary tracts [10,11,12]. Furthermore, considerable importance is given to proteins extracted from *Pisum sativum* thanks to its beneficial properties [13,14,15]. Pea protein may exercise bioactivities properties, including angiotensin I-converting enzyme inhibitor activity and antioxidant activity [13].

Xyloglucan (XG) is a hemicellulose of vegetable origin, taken from Tamarind tree seed (*Tamarindus indica*). Its properties on the intestinal mucosa are well known; in fact, it improves its resistance to pathological aggression and helps restore normal barrier functionality. It has been recently adopted in Europe as an active molecule for the restoration of physiological intestinal functions [16].

Moreover, XG has been formulated to control and reduce symptoms related to diarrheal events, such as abdominal tension and frequent faecal emissions, of different etiologies. Thanks to its polysaccharide configuration, XG gives the product a “mucin-like” molecular structure, forming a bioprotective film that improves resistance of mucosae to pathological aggression and helps to restore its normal function [16]. Furthermore, an in vitro experiment on epithelial cells of the bladder, infected with *Escherichia coli*, showed that XG was able to create a protective physical barrier that reduced urinary tract infection [10,11,16,17].

Pea protein (PP) is a protein derived from the plant of *Pisum sativum*. PP is a legume rich in fiber, low in fat, and boasting an extraordinary aminoacidic profile, especially rich in lysine, a key amino acid in the synthesis of collagen and carnitine [18]. Both compounds, given their protective properties towards mucosal barriers, could be effective in treating epidermis-related diseases.

Therefore, the aim of this study was to evaluate the effects of the association of XG + PP in an AD-induced mouse model as well as AD followed by a superinfection by *S. aureus.*

## 2. Results

### 2.1. Effect of Xyloglucan and Pea Protein on Histological AD Damage and Erythema Index

Histological examination of skin revealed characteristic pathological changes after oxazolone treatment and *S. aureus* infection. Xyloglucan and pea protein topic treatment significantly (*p* < 0.001) reduced the degree of tissue injury.

**Figure 1:**
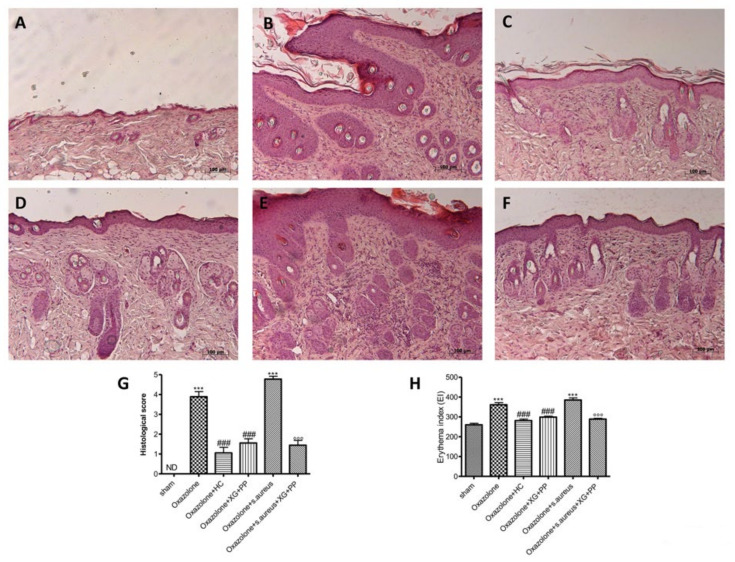
Effects of xyloglucan (XG) and pea protein (PP) on histological damage and erythema index induced by oxazolone and *S. aureus*: No histological variations have been found in the epidermis tissue collected from sham-operated mice (**A**) (see histological score). An extensive damage to the epidermis was evaluated in oxazolone-treated mice (**B**) (see histological score) and in oxazolone + *S. aureus* treated mice (**E**) (histological score) stained with hematoxylin–eosin (H&E). Hydrocortisone administration reduced the inflammation caused by oxazolone treatment (**C**) (histological score). Also, XG + PP topic treatment reduced significantly the severity of histological damage induced by oxazolone (**D**) (see histological score) and by oxazolone + *S. aureus* (**F**) (histological score). The picture is demonstrative of at least three experiments executed on distinctive experimental days. The histological score was made by an independent observer. The mice that received the administration of oxazolone and oxazolone + *S. aureus* showed a marked increase in erythema; instead, mice that received hydrocortisone administration and XG + PP by topical administration for both *in vivo* models revealed evidently reduced erythema (**H**). *** *p* < 0.001 vs. sham; ### *p* < 0.001 vs. oxazolone; °°° *p* < 0.001 vs. oxazolone + Staphylococcus. ND, not detectable.

The group treated with oxazolone (Figure 1B) and oxazolone + *S. aureus* (Figure 1E) showed evident hyperkeratosis and marked epidermal thickening. However, mice pretreated with xyloglucan and pea proteins for 4 weeks reduced epidermal thickening and inflammatory cell infiltration (Figure 1D,F). These results indicate that the administration of XG + PP could effectively inhibit the development of dermatitis in mice. The histological score was made by an independent observer. This data was confirmed by the erythema index where oxazolone and OX+ *S. aureus* prominently increased erythema, while mice that received XG and PP by topic administration revealed markedly reduced erythema (Figure 1H).

### 2.2. Effect of Xyloglucan and Pea Protein on Mast Cell Degranulation Induced by Skin Inflammatory Response

Mast cells affect the functions of keratinocytes and dendritic cells, inducing the expression of adhesion proteins, proinflammatory cytokines, and chemokines, as well as growth factors [19,20].

**Figure 2:**
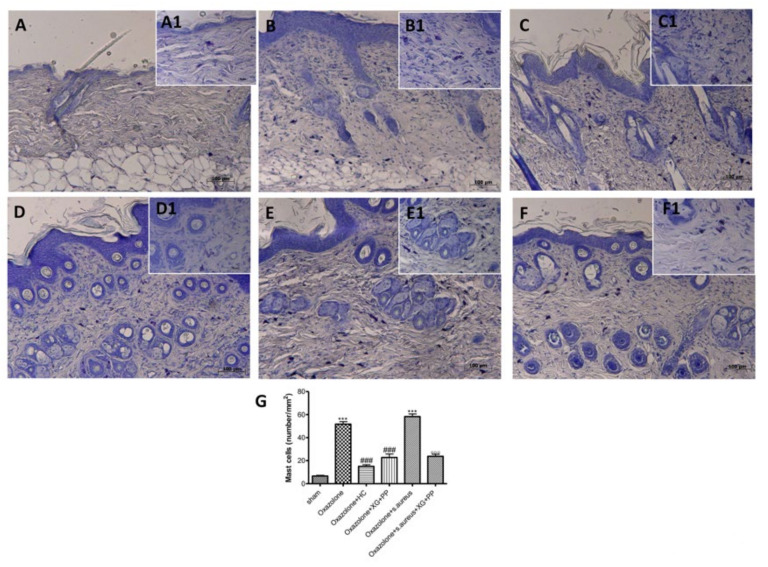
Effects of xyloglucan and pea protein on mast cell infiltration induced by oxazolone and *S. aureus:* An increased number of mast cells were detected in tissues from vehicle-treated mice (**B**,**G**; **E**,**G**) compared to the control group (**A**,**G**). Hydrocortisone administration reduced the number of mast cells (**C**,**G**). XG + PP-treated Atopic dermatitis (AD) mice showed fewer cells of this type (**D**,**F**,**G**). (**A1**–**F1**) A1-F1 depict the high magnification of the panels (40x) *** *p* < 0.001 vs. sham; ### *p* < 0.001 vs. oxazolone; °°° *p* < 0.001 vs. oxazolone + Staphylococcus.

Treatment with oxazolone (Figure 2B,B1) and oxazolone + *S. aureus* (Figure 2E,E1) induced increase in mast cell degranulation as determined by toluidine blue staining. Xyloglucan and pea protein significantly reduced the degranulation of mast cells for both *in vivo* models (Figure 2D,D1 and F,F1 respectively).

### 2.3. Effect of Xyloglucan and Pea Protein on Tight Junctions (TJ) and Filaggrin

Tight Junction (TJ) proteins have been shown to act as a barrier within the skin; the reduction of their expression causes an alteration in skin barrier functionality, determining an increase of allergen absorption leading to the activation of the immune system and inflammation. An intensification in the number of penetrating Langerhans cells in the TJ has been observed in AD, which may contribute to an increased absorption of allergens [21].

**Figure 3:**
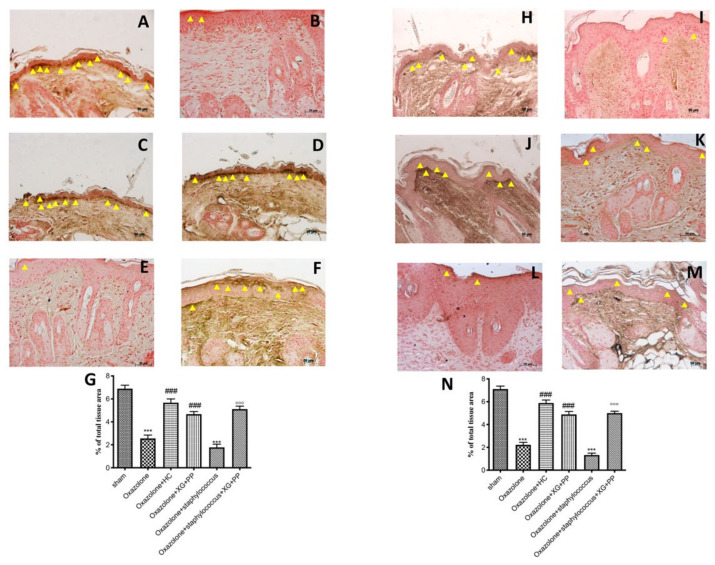
Effects of xyloglucan and pea protein on ZO-1 and occludin expressions: The ZO-1 and occludin expressions were analyzed using immunohistochemistry. Basal expressions of ZO-1 (**A**,**G**) and occludin (**H**,**N**) have been found in the tissues of sham-operated mice; oxazolone treatment reduced these expressions (**B**,**G**; **I**,**N**), whereas hydrocortisone administration (2.5 mg/mice) restored the expressions to almost basal levels in both *in vivo* models (**C**,**G** and **J**,**N** respectively). Also, XG + PP treatment increased the positive staining in oxazolone treatment and in oxazolone + *S. aureus* infection for both ZO-1 (**D**,**G** and **F**,**G** respectively ) and occludin (**K**,**N** and **M**,**N** respectively). Moreover superinfection induced by coadministration of oxazolone and *S. aureus* significantly reduced the positive staining of ZO-1 (**E**,**G**) and occludin (**L**,**N**). *** *p* < 0.001 vs. sham; ### *p* < 0.001 vs. oxazolone; °°° *p* < 0.001 vs. oxazolone + Staphylococcus.

Oxazolone treatment (Figure 3B,I) and *S. aureus* infection (Figure 3E,L) induced an increase of TJ permeability throughout the entire skin, whereas a significant reduction of the alteration of zonula occludens-1 (ZO-1) and occludin localization by immunohistochemistry was observed in XG and PP-treated mice (Figure 3D,F,K,M).

**Figure 4:**
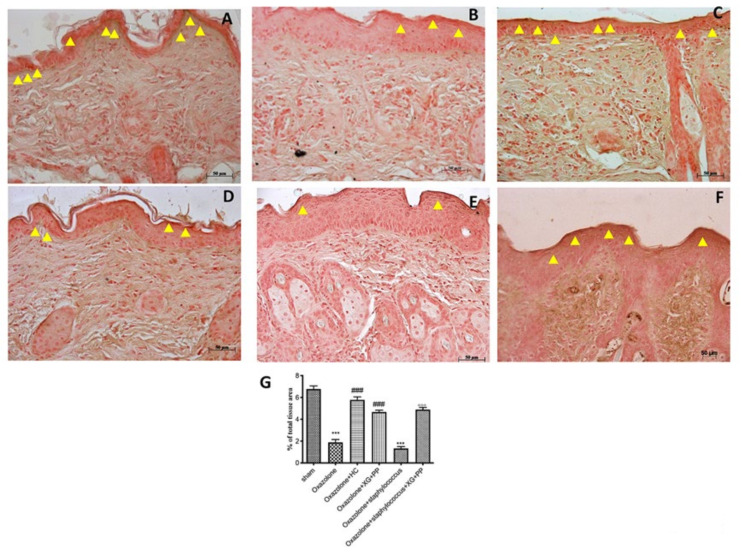
Effects of xyloglucan and pea protein on filaggrin expression: Immunohistochemical analysis of filaggrin was performed. A marked reduction of filaggrin was detected in the epidermal tissue of mice that received oxazolone (**B**,**G**) and oxazolone + *S. aureus* treatments (**E**,**G**) compared to sham-operated mice (**A**,**G**). Filaggrin expression was restored to near-endogenous levels by administration of hydrocortisone (**C**,**G**) and with XG + PP treatment in both *in vivo* models (**D**,**G** and **F**,**G** respectively). *** *p* < 0.001 vs. sham; ### *p* < 0.001 vs. oxazolone; °°° *p* < 0.001 vs. oxazolone + Staphylococcus.

Filaggrin is a key protein that supports the differentiation and formation of the skin barrier [22]. XG and PP were able to restore filaggrin positive staining (Figure 4D,F) compared to oxazolone (Figure 4B) and oxazolone + *S. aureus* treatments (Figure 4E).

### 2.4. Effects of Xyloglucan and Pea Protein on Inducible Nitric Oxide Synthase (iNOS) against Oxazolone and Oxazolone + S. Aureus Superinfection

**Figure 5:**
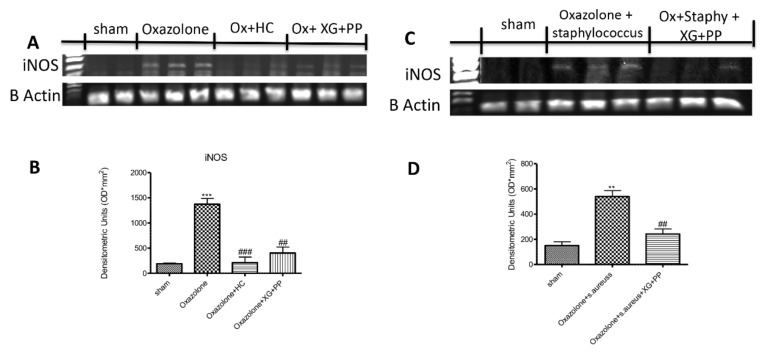
Effects of xyloglucan and pea protein on inducible nitric oxide synthase (iNOS) against oxazolone and oxazolone + *s. aureus* superinfection: The expression of iNOS mRNA was increased in mice of vehicle groups that received oxazolone treatment and oxazolone + *S. aureus* compared to the sham-operated mice. Moreover, XG + PP treatment and hydrocortisone administration significantly decrease iNOS mRNA levels (**A**,**B**). Furthermore, the administration of XG + PP significantly reduces the iNOS mRNA level in animals that underwent oxazolone + *S. aureus* superinfection (**C**,**D**). *** *p* < 0.001 vs. sham; ** *p* < 0.01 vs. sham; ### *p* < 0.001 vs. oxazolone; ## *p* < 0.01 vs. oxazolone; ## *p* < 0.01 vs. oxazolone + Staphylococcus.

Inducible nitric oxide synthase is involved in atopic dermatitis flare-ups [23]. A significant increase in inducible nitric oxide synthase (iNOS) (500 bp) mRNA expression following oxazolone and *S. aureus* topic administration was evident in the lesioned skin of mice (Figure 5A,C). Moreover, xyloglucan and pea protein significantly decrease iNOS mRNA levels (Figure 5A,C, see densitometry analysis Figure 5B,D respectively).

### 2.5. Effects of Xyloglucan and Pea protein on Cytokin Production Following Oxazolone and Oxazolone + S. Aureus Superinfection.

**Figure 6:**
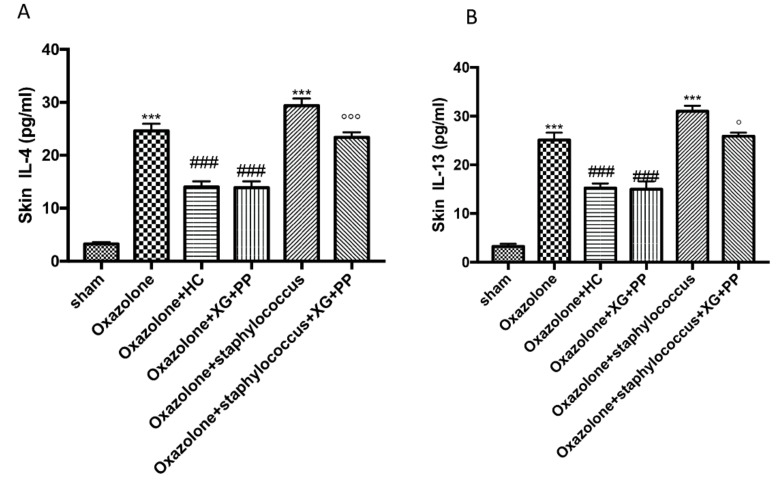
Effect of xyloglucan and pea protein on Interleukin (IL)-4 and IL-13 expressions: The levels of IL-4 (**A**) and IL-13 (**B**) were significantly increased in mice treated with oxazolone. The increases in levels of IL-4 and IL-13 were significantly attenuated in the mice receiving XG and pea protein topical administration (**A**,**B**, respectively). *** *p* < 0.001 vs. sham; ### *p* < 0.001 vs. oxazolone; °°° *p* < 0.001 and ° *p* < 0.05 vs. oxazolone + Staphylococcus.

Interleukin (IL)-4 and IL-13 are potent mediators of type 2-associated inflammation typically found in AD-lesioned skin. IL-4 shares its biological functions with IL-13, a finding that is explained by their ability to signal via the type 2 IL-4 receptor (R), which is composed of IL-4Rα in association with IL-13Rα1 [24]. A substantial increase of IL-4 and IL-13 levels was observed in skin tissues collected from both oxazolone and *S. aureus* treatment (Figure 6A,B, respectively). In contrast, treatment with the association of XG + PP significantly reduced cytokine levels in both models (Figure 6A,B, respectively).

## 3. Discussion

AD is characterized by a complex pathogenesis that involves genetic susceptibility and immunological and epidermal barrier dysfunction [25]. Intense itching and frequent exacerbations are significant burdens for patients with AD, leading to impaired quality of life, especially in patients with extensive lesions [26,27]. 

Bacterial infections are very common, particularly due to Staphylococcus and Streptococcus strains which contribute to erythema aggravation [28,29,30]. Currently, pharmacological treatments involve the use of corticosteroids and antihistamines, while the most serious cases require the use of immunomodulating agents [31,32]. Thus, the research of new molecules that can represent a helpful therapy for conventional drugs is certainly a great resource that should not be underestimated.

Presently, great attention is focused on substances of natural origin, namely XG and PP, two compounds defined as “mucosal protectors”. On intestinal mucosa, it has been shown that these products are able to form a bioprotective film, preventing contact with pathogens and their toxins, lipopolysaccharide (LPS), etc.; thanks to the layer formation on the intestinal mucosa, they increase resistance to pathological aggressions, helping to restore normal mucosal function [14,33,34]. Moreover, recent studies have shown the efficacious protective role of XG in bacterial infections as well as in urinary tract infections [11,17].

Based on the XG and PP properties, the aim of our study was to evaluate these compounds in a cream mixture in order to examine the protective activity on the epidermis in experimental models of AD and AD followed by *S. aureus* infection in mice. *S. aureus* is a gram-positive bacterium, and it is the most frequent cause of skin infections [35]. Likewise, in AD, we assist a decreased microbial diversity in favor of an increased amount of Staphylococcus species [36]. More than 85% of AD patients are colonized by facultative anaerobic pathogenic bacteria such as *S. aureus*, which is strongly associated with an increase in disease severity [37]; particularly *S. aureus*, through upper corneocytes, can penetrate the epidermis via the intercellular spaces, probably related to lipid deficiencies in AD skin [38].

It was well studied that, upon treatment with oxazolone, engrafted mice developed specific characteristics which included epithelial hyperplasia, mast cells infiltration into the dermis and epidermis, and IgE secretion [39]. Mast cells, as an important component of innate immunity, have been observed in AD skin lesions [40]. In fact, mast cells are strategically sited at the interface between the external environment and the epithelium [41,42,43]. In response to epithelial insults, mast cells are an active player in wound healing and production of cytokines [44].

Our results clearly showed significant epidermal morphological changes following AD induction and *S. aureus* infection, which was characterized by epidermal thickness with hyperkeratosis, increasing erythema and degranulation of the mast cells. The treatment with XG + PP significantly reduced all the histological changes that characterized AD.

It is important to emphasize that the AD is characterized not only by a greater immune reaction but also by barrier defects; in fact, AD patients show a disturbed inside-out barrier.

TJs are cell–cell junctions important to the foundation for the paracellular barrier in the skin [45]. They are able to control paracellular diffusion of water, solutes, and pathogens [46]. On one hand, their central localization guarantees the interaction between the stratum corneum and the microbiome barrier; on the other hand, they interact with the immunological and chemical barriers [22,47,48,49]. Moreover, it has been showed that *S. aureus* can reduce the levels of TJ proteins in the AD skin [50]. In fact, *S. aureus* can worsen barrier impairment by releasing proteases, enzymes, and cytolytic toxins that induce cell injuries [51].

According to these scientific evidences, our results suggested that the treatment with XG + PP had the ability to modulate the loss of dermal permeability induced by oxazolone, promoting a substantial increasing of TJ expression.

Among the fundamental proteins for the integrity of the skin in patients affect by AD, we also examined filaggrin. Filaggrin is a key protein in the maintenance and differentiation of the skin barrier [52]; specifically, it is an important protein because it plays an important role in the formation of the cornified cellular envelope, essential for the homeostasis of the skin barrier [53]. Moreover, mutations and deficiencies of the filaggrin gene have been recently recognized as an important risk factors for the development of AD [54]; *de facto*, about one third of European AD patients show a filaggrin genetic mutation [55,56,57]. Filaggrin-deficient patients show a significant increase in surface pH [58], which is part of the complex barrier system of the skin [59], and this condition could facilitate bacterial growth [60]. Our results clearly showed a significant decrease of filaggrin expression following *AD* induction and *S. aureus* infection, confirming its main role in the pathogenesis of AD. Conversely, following XG + PP treatment, we assisted in a significant downregulation of filaggrin expression compared to AD mice that was confirmed also in the superinfection model. During inflammatory stimuli, dermal endothelial cells or infiltrating cells, closely associated with the endothelium, produce iNOS, one of three isoforms belonging to the family of nitric oxide synthases [61]; consequently, iNOS produces a large amount of NO. It has been demonstrated that iNOS has a key role in the modulation of symptoms in patients with inflammatory diseases, including AD, specifically it increases scratching and immunoglobulin E release (IgE) in plasma [23]. This study indicated that the synergistic administration of XG + PP had the ability to reduce iNOS expression in both AD models. The increased levels of cytokines, IgE production, and histamine release are closely associated to inflammation in AD [62]. In particular, IL-4 and IL-13 are highly expressed in the lesioned skin and cover a key role as regulators of different features of AD like epidermal hyperplasia, skin barrier dysfunction, and production of eosinophil [24]. The levels of cytokines were significantly attenuated in the mice receiving XG + PP topical application.

The results of our study showed that XG, in association with PP, offers a new way to protect the epidermis. In particular, the efficacy of XG associated with PP is related to the modulation of the erythema formation, mast cell infiltration, TJs, and filaggrin in the skin. Furthermore, this association can help to modulate the infection of bacterial colonies by reducing the frequent events in AD patients. In conclusion, we demonstrated the non-pharmacological barrier properties of XG and PP on a preclinical model of AD that could represent a promising alternative approach to common drugs for AD; therefore, the use of a product based on natural compounds could prevent and help the patient in managing the disease and the related symptoms by reducing side effects and by improving the quality of life.

## 4. Materials and Methods

### 4.1. Materials

XG and PP were mixed in a neutral cream to have a final concentration of 5% (kindly provided by DEVINTEC SAGL (Lugano, Switzerland). All other chemicals were obtained from the highest grade of commercial source. Oxazolone and hydrocortisone were purchased by Sigma-Aldrich Company Ltd. (Milan, Italy). *S. aureus* (ATCC 29213) was purchased by (American Type Culture Collection) ATCC materials resource (Manassas, Virginia, VA, USA).

### 4.2. Animals 

Specific pathogen-free 5-week-old female SKH-1 hairless mice (Envigo, Milan, Italy) were accommodated in an accurate environment (22 ± 2 °C, 55% ± 15% relative humidity, 12 h light/dark cycle) and fed with a standard diet and water. The acclimation was made for one week. This study was approved by the University of Messina Review Board for the care of animals in compliance with Italian regulations on protection of animals (n° 96/2016-PR released on 02/02/2016, art. 31 of D.lgs. 26/2014) and other scientific purposes (DM 116192) as well as with EU regulations (OJ of EC L 358/1 12/18/1986).

### 4.3. Staphylococcus aureus (SA) Culture

For the experimental infection, the *S. aureus* strain was grown to the exponentially phase (about 1 × 10^9^ CFU/mL) in brain heart infusion (BHI) broth at 37 °C overnight with shaking and harvested by centrifugation (5000× *g* for 5 min), washed (3× in PBS), and suspended to the required number in PBS. The viable count to reach the needed number was made through a spread plate technique.

### 4.4. Induction of AD-Like Skin Lesions and Sample Treatment

The animals were topically treated with 200 µL of oxazolone at 0.5% (Sigma-Aldrich, St. Louis, MO, USA) to the dorsal skin three times a week for a period of two weeks. Induction of AD was confirmed through macroscopic observation (e.g., eruption and erythema). Forty-eight mice were divided into six experimental groups:

### 4.5. Experimental Groups

Group 1: mice received vehicle (polysorbate 80) without oxazolone treatment for 4 weeks (*n* = 8)
Group 2: mice received vehicle with oxazolone treatment (5 mg/mL) for 4 weeks (*n* = 8)
Group 3: mice received XG and PP (topical administration) 1 h before oxazolone treatment for 4 weeks (*n* = 8)
Group 4: mice received hydrocortisone (HC; 2.5 mg/mice) with oxazolone treatment for 4 weeks (*n* = 8)
Group 5: mice received vehicle with oxazolone treatment for 4 weeks and superficial skin superinfection was induced by placing on the skin a 5-μL droplet containing 10^8^ cells concentrated from an overnight Staphylococcus *aureus* bacterial culture for 2 weeks (*n* = 8)
Group 6: mice received XG and PP 1 h before oxazolone treatment plus skin superinfection by *S. aureus* (*n* = 8)

Mice, except those in group 1 were topically treated with 0.5% oxazolone (200 µL) to the dorsal skin three times a week for 4 weeks (12 challenges totally) [63]. Mice in groups 3 received XG and PP and group 4 received hydrocortisone, both daily for 4 weeks using oral feeding needles in addition to topical oxazolone treatment. Group 5 received *S. aureus* inoculation in addition to oxazolone 2 weeks following the 2 weeks of only oxazolone [64]. Group 6 received XG + PP together with the starting of superinfection. At the end of the 4-week oral administration period, erythema was measured. After the experiments, animals were sacrificed, some skin lesions were removed and stored at −80 °C until biochemical analysis, and some were fixed with 10% neutral formalin for histological analyses and immunohistochemical localization.

### 4.6. Histological Examination

To identify epidermal thickness and inflammatory cells infiltration, sections were stained with hematoxylin–eosin (H&E). Histological evaluations were performed as previously described by Reference [11].

After dehydration in graded ethanol and xylol, the tissues were embedded in paraffin and cut to the microtome in order to obtain sections of 7 μm thickness. Tissue sections, after being deparaffinized in xylol and rehydrated through a descending scale of ethanol, were stained with hematoxylin–eosin (H&E, Bio-Optica, Milano, Italy) and examined with an optical microscope (Axostar Plus equipped with Axio-Cam MRc, Zeiss, New York, NY, USA) to observe the structure of the epidermis. The histological results were shown at 10× (100 μm of the bar scale).

### 4.7. Toluidine Blue Staining

Epidermis tissue sections were stained with toluidine blue to assess the number of mast cells and their degranulation. Sections were deparaffinized, sited in water for 5 min, and next relocated to toluidine blue for 4 min and then blotted carefully. Sections were placed for 1 min in absolute alcohol, cleared in xylene, and fixed on glass slides using Eukitt (Bio-Optica, Milan, Italy). The number of metachromatic stained mast cells was obtained by counting in five high-power fields (40×) per section by using an Axiovision Zeiss (Milan, Italy) microscope-correlated software (Carl Zeiss Vision, Jena, Germany).

### 4.8. Localization of Filaggrin, Occludin, and ZO-1 by Immunohistochemistry Analysis

Immunohistochemical localization was performed as previously described [11]. Slides were incubated overnight in a room with room temperature, using rabbit polyclonal primary antibodies: anti-filaggrin (Abcam, 1:200 in PBS, *v*/*v*, Cambridge, UK), anti-occludin (Invitrogen, 1:200 in PBS, *v*/*v,* Carlsbad, CA, USA), and anti-ZO1 (Invitrogen, 1:200 in PBS, *v/v,* Carlsbad, CA, USA). At the end of the incubation with the primary antibody, the sections were abundantly washed with PBS and incubated with a secondary antibody (Santa Cruz Biotechnology, Santa Cruz, CA, USA) for 1 h. All stained sections were observed with an Inverted Microscope with twin charge-coupled device (CCD) cameras (magnification, ×200; Nikon, Tokyo, Japan).

For the graphical display of densitometric analyzes, the percentage of positive staining was measured using a computerized image analysis system (Leica QWin V3, Cambridge, UK). The images were acquired using an optical microscope (Zeiss, Axio Vision). For immunohistochemistry, the images were shown at a magnification of 20× (50 μm of the bar scale).

### 4.9. Semiquantitative Reverse Transcriptase-Polymerase Chain Reaction (RT-PCR)

Total RNA isolated from the skin was obtained with Trizol reagent (Invitrogen, Carlsbad, CA, USA) according to the manufacturer’s protocol and quantified by spectrophotometry at 260 nm. Reverse transcription (RT) was made as previously described [65]. Oligonucleotide primers specific for mouse iNOS and β-actin are shown in Table 1.

### 4.10. ELISA Assay for IL-4 and IL-13

The skin tissue was thawed on ice and homogenized in 300 μL lysis buffer (750 μL, Pierce #87787, Thermo Fisher Scientific, Waltham, MA, USA) supplemented with a protease inhibitor cocktail (Sigma-Aldrich, Rehovot, Israel). Thereafter, the samples were homogenized and centrifuged at 14,000× *g* for 10 min at 4 °C; supernatants were collected, aliquoted, and stored at −20 °C. Cytokines were measured by ELISA according to the manufacturer’s instructions. The following kits for mouse proteins were used: IL-4 (BioLegend, San Diego, CA, USA) and IL-13 (R&D Systems, Minneapolis, MN, USA).

### 4.11. Statistical Analysis

All values are showed as mean ± standard error of the mean (SEM) of *N* observations. *N* denotes the number of animals employed. The experiment is demonstrative of at least three experiments performed on different days on tissue sections collected from all animals in each group. Data were examined by one-way ANOVA followed by a Bonferroni post hoc test for multiple comparisons. A *p*-value of less than 0.05 was considered significant.

## Figures and Tables

**Table 1:** Primers used for RT-PCR.

GENE	Primer-Sequence	Product Length (bp)
Mouse iNOS	Forward 5′- GCCTCGCTCTGGAAAGA -3′Reverse 5′-TCCATGCAGACAACCTT -3′	500
Mouse β-actin	Forward 5′-TAA CCA ACT GGG ACG ATA TG-3′Reverse 5′-ATA CAG GGA CAG CAC AGC CT-3′	203
